# Inhibitory effect of the combination therapy of simvastatin and pinocembrin on atherosclerosis in apoE-deficient mice

**DOI:** 10.1186/1476-511X-11-166

**Published:** 2012-12-05

**Authors:** Hui Sang, Na Yuan, Shutong Yao, Furong Li, Jiafu Wang, Yongqi Fang, Shucun Qin

**Affiliations:** 1 Institute of Atherosclerosis, Key Laboratory of Atherosclerosis in Universities of Shandong, Taishan Medical University, Taian, 271000, China; 2 College of Basic Medical Sciences, Taishan Medical University, Taian, 271000, China; 3 School of Pharmacy, Taishan Medical University, Taian, 271000, China

**Keywords:** Pinocembrin, Simvastatin, Combined therapy, Atherosclerotic lesion, Apolipoprotein E knockout mice

## Abstract

The present study was performed to investigate the effects of the combination therapy of pinocembrin and simvastatin on the atherosclerotic lesions development in the ApoE^−/−^ mice.

**Methods:**

Eight-week-old male ApoE^−/−^ mice were fed high fat diet (HFD) and treated with simvastatin (10 mg/kg per day), pinocembrin (20 mg/kg per day), or the combination therapy (simvastatin 5 mg/kg per day and pinocembrin 20 mg/kg per day) for 14 weeks. The serum lipid levels, nitric oxide (NO), endothelin (ET), superoxide dismutase (SOD) and malondialdehyde (MDA) were determined with spectrophotometric measurement and ELISA assay. Vascular endothelial growth factor (VEGF) in serum and aortic root was detected. En face analyses of atherosclerotic lesion in whole aorta and aortic root sections were performed with plaque staining using oil red O.

**Results:**

The combination treatment with simvastatin and pinocembrin resulted in significantly decreased levels of serum total cholesterol, triglycerides and low-density lipoprotein cholesterol, augmented NO levels and SOD activity, inhibited ET and VEGF expression. Immunohistochemistry of aortic valve sections revealed that the combination therapy also suppressed the expression of VEGF induced by HFD. In addition, HFD-induced arterial wall lipid disposition displayed by oil red O staining was reduced significantly in aortic root and whole aorta en face in the combination administrated mice. The effect of the combination was superior to simvastatin alone.

**Conclusion:**

The combination of simvastatin and pinocembrin synergistically inhibited atherosclerotic lesion development in ApoE^−/−^ mice with hyperlipidemia, which is partially dependent on the protective of vascular endothelium.

## Background

Atherosclerosis is the primary underlying cause of cardiovascular disease and the major cause of mortality in the western world today [1]. Atherogenic stimuli, including dyslipidemia and oxidative stress, induce vascular endothelial dysfunction which is considered as an early marker for atherosclerosis [2]. Vascular endothelial cells are not merely constituents of the vessel wall but are able to respond to physiological stress, which play important roles in the maintenance of vascular integrity including the regulation of vascular tone, vascular permeability, vessel wall inflammation, and thrombosis [3]. Endothelial dysfunction, which occurs in dyslipidemia, has been identified as a common link of all cardiovascular risk factors. The hallmark of endothelial dysfunction is impaired endothelium dependent vasodilation, which is mediated by nitric oxide (NO). A number of clinical studies have demonstrated that impaired NO-dependent vasodilatation is closely related to atherosclerosis [4]. Thus, protecting vascular endothelial cells is an attractive strategy to combat atherosclerotic lesion progression.

Lipid-lowering interventions are the cornerstone for the prevention and treatment of atherosclerotic disease. Statins lower cholesterol levels by inhibiting 3-hydroxy-3- methyl-lutaryl coenzyme A (HMG-CoA) reductase, which is the rate-limiting enzyme of the mevalonate pathway of cholesterol synthesis. Large-scale clinical studies have demonstrated that statins treatment reduces the relative risk for cardiovascular disease and stroke in hypercholesterolemic patients. However, a long-term and high-dose application of simvastatin can increase certain side effects, such as myopathy and liver damage [5]. In addition, high-risk patients on statins treatment continue to have high risk for future cardiovascular events [6]. Thus, lower doses of combinatorial therapy may render better efficacy and increased safety compared to high doses of the single agents. It is unclear what type of drugs could be combined with statins to provide the enhanced effects [7].

Propolis is a sticky, resinous and dark-colored natural substance produced by honeybees (Apis mellifera) and has been used as a folk medicine in many countries from ancient times. Propolis has been reported to have physiological functions such as antibacterial, anti-viral, anti-inflammatory, anti-oxidative and anti-carcinogenesis activities [8]. The chemical constituent of propolis is extremely complex and its flavonoid derivatives have been widely cited as its biologically active compounds. Pinocembrin, the most abundant flavonoid monomer in propolis [9], has anti-inflammatory, antioxidant, lowering blood lipids and protecting vascular endothelial cells properties [10]. However, whether pinocembrin combinating with simvastatin inhibits synergistically atherosclerotic lesion development remains unclear. In the present study, the combining therapy of pinocembrin and simvastatin was administered to 8-week-old apoE^−/−^ mice fed high fat diets, and changes in serum lipid and endothelial function were evaluated over 14 weeks of treatment. The data demonstrate that combining therapy reduces serum lipid, protects vascular endothelial cells from dietary cholesterol–induced dysfunction and inhibits the development of atherosclerosis lesion in apoE^−/−^ mice.

## Materials and methods

### Materials

Vascular endothelial growth factor (VEGF) and endothelin (ET) enzyme-linked immunosorbent assay (ELISA) kits were purchased from R&D Systems Inc (Minnesota, USA). Oil red O was obtained from Sigma Chemical Co (St. Louis, MO, USA). Antibody against VEGF was provided by Santa Cruz Biotechnology (Santa Cruz, CA, USA). Assay kits used for total cholesterol (TC), triglyceride (TG), low-density lipoprotein cholesterol (LDL-C) and high-density lipoprotein cholesterol (HDL-C) were from Biosino Bio-Technology & Science INC (Beijing, China). The assay kits for superoxide dismutase (SOD), malondialdehyde (MDA) and nitric oxide (NO) were purchased from the Nan-jing Jiancheng Bioengineering Institute (Nanjing, China).

### Isolation and identification pinocembrin

**Figure 1:**
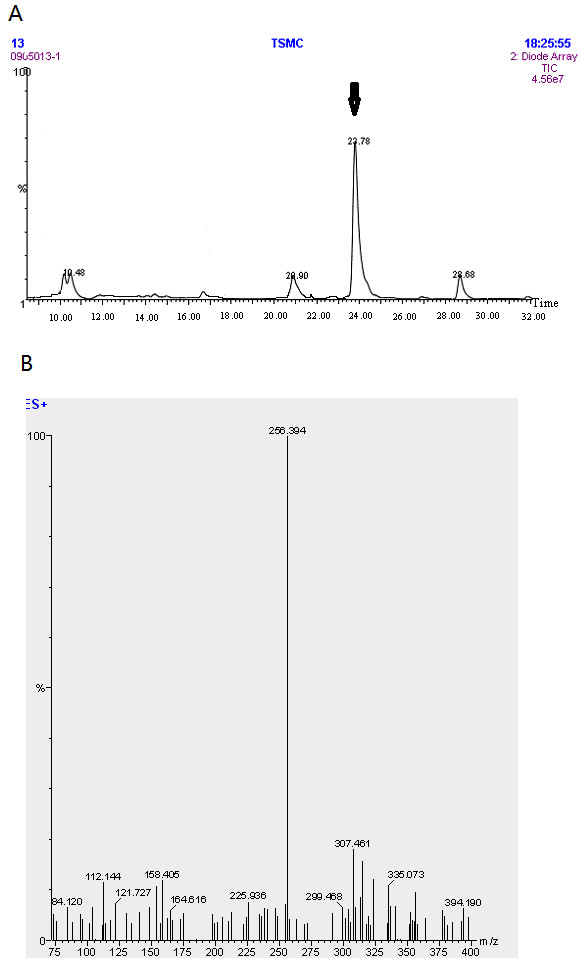
**Identification of pinocembrin in propolis by MS analysis.A**. Purify pinocembrin from the crude flavonoids of propolis. The retention time of the peak (the black arrow) was consistent with standard pinocembrin, and its purity reached 91.1% as determined by HPLC. **B**. The peak (the black arrow) on HPLC gave a major molecular ion [M + H]^+^ at m/z 256.394, which was consistent with standard pinocembrin.

Crude propolis from Taishan in China was frozen at −18°C and ground into powder. The powder (100 g) was dissolved in 70% ethanol (w:w = 1:4) at room temperature for 24 h and oscillated ultrasonically for 35 min at 35°C constant temperature, and then filtered after 2 h. The solvent of the supernatant was evaporated under reduced pressure to produce the ethanol extract of propolis (EEP, 65 g). EEP was put into a silica gel column (200–300 mesh), eluted and concentrated, and crude flavonoids (52 g) were subsequently obtained. The crude flavonoids were separated into several fractions by high performance liquid chromatography (HPLC) according to a previously described method [11]. These fractions were further investigated by the mass spectrometry (MS). MS analysis indicated that the compound (colorless minute needles, mp 194–197) was pinocembrin (5,7-dihydroxy-flavanone, 1.8 g, purity 92%) (Figure 1A and B). Pinocembrin was used for further experiments.

### Experimental animals

Male ApoE^−/−^ mice on a C57BL/6 J background were purchased from the Laboratory Animal Center of Peking University (Beijing, China). All experiments were approved by the Laboratory Animals’ Ethical Committee of Taishan Medical University and followed national guidelines for the care and use of animals. ApoE^−/−^ mice (8 weeks old) were randomly allocated to four groups receiving a high fat diet (21% fat and 0.15% cholesterol) together with simvastatin (10 mg/kg·d), pinocembrin (20 mg/kg·d), a combination (simvastatin 5 mg/kg·d and pinocembrin 20 mg/kg·d), or vehicle for 14 weeks.

### Histology and immunohistochemistry

Following 14 weeks treatment, the mice were anesthetized with an intraperitoneal injection of sodium pentobarbital (40 mg/kg) and blood was collected from the retro-orbital sinus of the mice after fasting for 12 h. The mice were sacrificed by exsanguinations and perfused with 10 ml ice-cold PBS at physiological pressure via the left ventricle. The aortas were isolated and the adventitia was thoroughly cleaned under a dissecting microscope, then cut opened longitudinally and stained with oil red O. The percentage of the plaque area stained by oil red O to the total luminal surface area was determined.

To further quantify the atherosclerotic lesions in the aortic root, serial cryostat sections (8 μm) were prepared as described [12]. In brief, atherosclerotic lesions in the aortic root were examined at 3 locations and each separated by 120 μm, 4 to 5 serial sections were prepared from each location. Some of the sections were stained with oil red O and the lipid composition of the lesion was determined by calculating the percent of the oil red O positive area to the total cross-sectional vessel wall area. The remaining sections were used for immunohistochemical analysis. Air-dried cryostat sections were fixed in acetone and stained with the VEGF antibody (1:200) using an immunohistochemical kit. The sections were then counterstained with hematoxylin. Nonimmune IgG was used as negative controls. Cells with fine yellow particles in the cytoplasm were defined as positive. All images were captured with a Olympus BX51 microscope equipped with a video camera and analyzed using Image–Pro-Plus 6.0 software (version 6.0, Media Cybernetics, MD, USA). In each case, the average value for 4 to 5 locations or sections of each animal was used for analysis.

### Serum analyses

Serum TC and TG were measured by enzymatic colorimetric assays, HDL-C levels were assessed by direct method, and LDL-C was calculated according to the Fridewald formula.

The content of NO and activity of SOD in serum were measured by nitric acid reductase method and xanthine oxidase method, respectively, and thiobarbituric acid reactive substance (TBARS) was adopted to determine serum MDA level. VEGF and ET in serum were determined by respective ELISA kits according to the manufacturer’s instructions.

### Statistical analysis

All data are presented as means ± SEM. Statistical analysis was performed using one-way analysis of variance (ANOVA) followed by Student–Newmann–Keuls multiple comparison tests with the SPSS 13.0 software for Windows. Probability values less than 0.05 were considered statistically significant.

## Results

### The combined treatment reduced serum lipid levels

**Figure 2:**
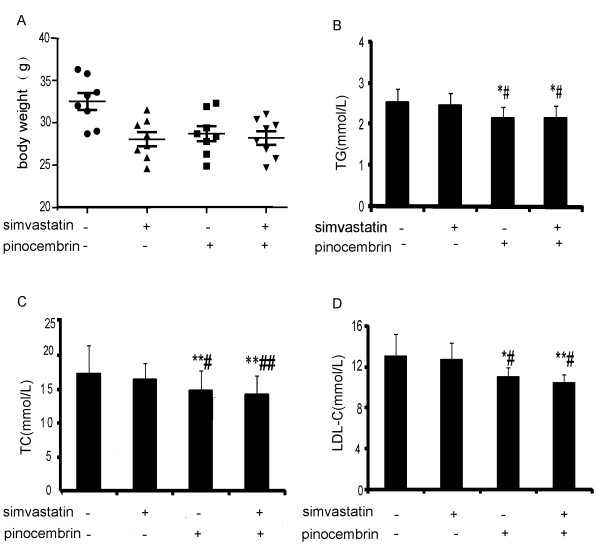
**The combination therapy inhibited the body weight gain and decreased serum lipid in the ApoE^−/−^ mice.** The ApoE^−/−^ mice were fed high fat diet and given vehicle alone, simvastatin (10 mg/kg), pinocembrin (20 mg/kg ), or the combination of simvastatin (5 mg/kg) and pinocembrin (20 mg/kg) per day for 14 weeks. **A**: body weight for each mouse was measured at the end of experiment. **B**-**D**: the levels of serum lipid. Data are presented as the mean ± SEM (n = 8). ^*^P < 0.05, ^**^P < 0.01 versus vehicle-treated group; ^#^*P* < 0.05, ^##^*P* < 0.01 versus simvastatin treatment.

High fat diet for 14 weeks induced weight gain and hyperlipidemia in the apoE^−/−^ mice. As shown in Figure 2A, the mean body weight of the mice accepted vehicle was 32.5 ± 2.81 g. However, drugs treatment significantly controlled mouse weight gain by 3–4 g. Mean body weight of combination therapy mice was 28.21 ± 2.26 g, whereas the final weights of mice undergoing simvastatin, pinocembrin, and combination therapy treatment did not differ grossly.

We did not expect simvastatin to influence serum lipid levels, given previous results in our laboratory [13] and others [14,15]. As shown in Figure 2B-D, simvastatin treatment for 14 weeks had no significant effect on serum TC, TG or LDL-C compared to those in vehicle-treated group, while the combination of simvastatin and pinocembrin significantly decreased TC, TG and LDL-C by 13.39%, 13.41% and 17.62%, respectively.

### The combination therapy improved vascular endothelial function in apoE^−/−^ mice

**Figure 3:**
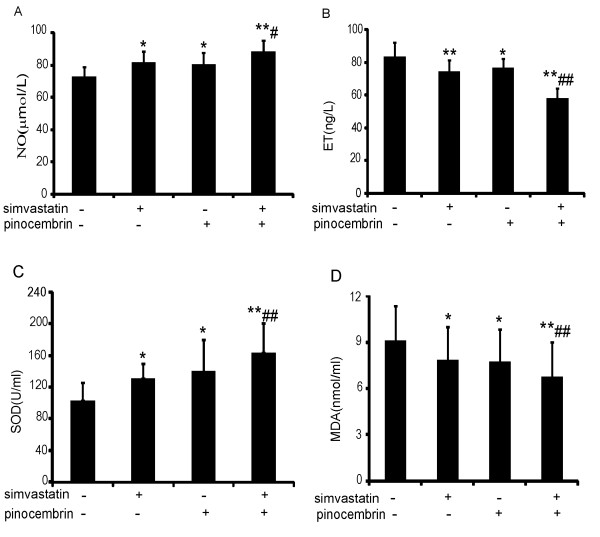
**Effect of the combination of simvastatin and pinocembrin on the levels of NO, ET, SOD and MDA in serum of apoE^−/−^ mice.** The mice were treated as in Figure 2 and the levels of NO (**A**), ET (**B**) , SOD (**C**) and MDA (**D**) in serum were measured as described as in materials and methods. Data are presented as the mean ± SEM (n = 8). ^*^P < 0.05, ^**^P < 0.01 versus vehicle-treated group; ^#^*P* < 0.05, ^##^*P* < 0.01 versus simvastatin treatment. SOD: superoxide dismutase, MDA: malondialdehyde, NO: nitric oxide , ET: endothelin.

Endothelial cells not only respond to but also produce and release vasoactive substances that relax or constrict blood vessels. Endothelial dysfunction is characterized by diminished production or bioavailability of NO and by alterations in other important vasoactive molecules, such as endothelin (ET), resulting in impaired endothelium-dependent vasodilation [2]. Stimulation of endothelial NO production has been suggested as an important endothelial protective effect. We investigated the serum concentration of NO and ET. As shown in Figure 3A and B, compared to vehicle-treated group, the effect of simvastatin on serum NO level was modest and similar to that of pinocembrin, but the combination of simvastatin and pinocembrin augmented significantly the NO level. Meanwhile, ET levels reduced in the animals treated with simvastatin, pinocembrin and the combination by 10.54%, 8.14% and 13.41%, respectively, when compared with the high fat diet only group.

### Effect of the combination therapy on serum MDA level and SOD activity

In the pathogenesis of hypercholesterolemia, excessive production of vascular ROS such as ·O_2_^-^ can reacts with NO, which not only results in loss of NO bioactivity, but also promotes formation of peroxynitrite anion (ONOO^-^). In the vessel wall, peroxynitrite and peroxynitrous acid may contribute to lipid peroxidation and membrane damage. SOD potentially plays an important role in protecting NO from superoxide anion in the extracellular space [16]. Therefore MDA level and SOD activity in serum were assessed (Figure 3C and D). Compared to vehicle-treated group, both single and combined therapy of simvastatin and pinocembrin significantly decreased the levels of MDA, and the combined therapy had the lowest MDA. Inversely, the combination therapy had the highest activity of SOD. These data indicated that the combination of simvastatin and pinocembrin substantially inhibited hypercholesterolemia-induced the production of MDA and increased SOD activity in the ApoE^−/−^ mice.

### The combination therapy reduced the expression of VEGF

**Figure 4:**
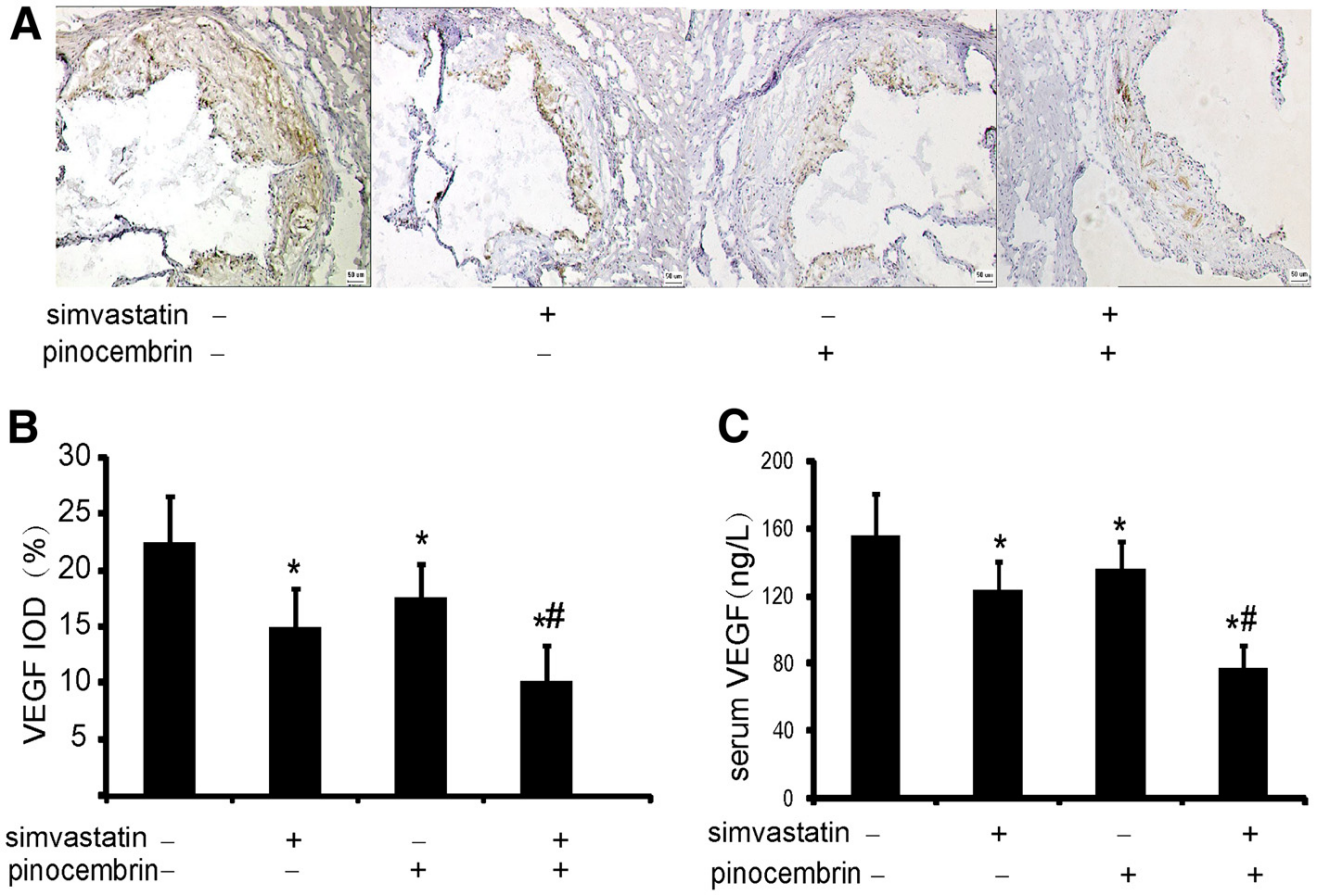
**The expression of VEGF on the aorta and in serum of apoE^−/−^ mice treated as in Figure**2. **A-B**: immunohistochemistry staining of aortic sections with VEGF antibody (blue = nuclei, brown = target protein) and integral optical density (IOD) values. **C**: the level of VEGF in serum of apoE^−/−^ mice. ^*^P < 0.05, ^**^P < 0.01 versus vehicle-treated group; ^#^*P* < 0.05versus simvastatin treatment.

Hypercholesterolemia increases oxidative stress and decreases NO bioavailability, which induces vascular endothelial growth factor (VEGF) upregulation in the vessel wall. VEGF not only promotes normal and pathological angiogenesis but also plays an important role in atherosclerotic lesions. VEGF is a chemotactic factor for macrophages and vascular smooth muscle cells, and induces synthesis of metalloproteinases and adhesion molecules [17]. In the present study, VEGF expression was detected by immunohistochemistry and ELISA. As shown in Figure 4A and B, drugs treatment significantly decreased the positive area of VEGF within the atherosclerotic lesion induced by high fat diet. Compared to vehicle-treated group, VEGF integral optical density (IOD) values decreased by 33.1%, 21.5%, and 54.5% in the simvastatin, pinocembrin, combination groups, respectively. Serum VEGF levels detected by the ELISA were consistent with the immunohistochemical findings (Figure 4C).

### The combined treatment decreased atherosclerotic lesions in the aorta of apoE^−/−^ mice

**Figure 5:**
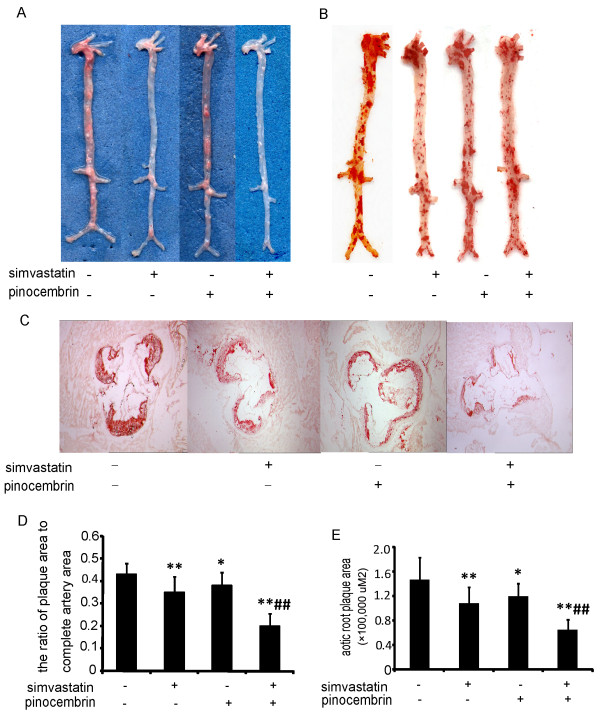
**The combination of simvastatin and pinocembrin decreased atherosclerotic lesions on the aorta in the apoE^−/−^ mice.A**: the gross appearance of the ascending aorta to the abdominal aorta. **B**: lipid accumulation staining using oil red O of longitudinally opened aorta in the apoE^−/−^ mice. **C**: aortic root plaque stained by oil red O in ApoE^−/−^ Mice. **D**: the ratio of the plaque area stained by oil red O to the total luminal surface area. **E**: the aortic root plaque area. Data are presented as the mean ± SEM (n = 8). ^*^P < 0.05, ^**^P < 0.01 versus vehicle-treated group; ^#^*P* < 0.05, ^##^*P* < 0.01 versus simvastatin treatment.

We investigated the effect of the combined treatment on the size of atherosclerotic lesions in apoE^−/−^ mice. As shown in Figure 5A, atherosclerotic lesions were distributed mainly in the aortic arch and the areas surrounding the branching points of the major arteries. The percent of the aortic lesion area was measured by quantitative histomorphology of the oil red O-stained en face specimens (Figure 5B and D). Compared to vehicle-treated group, the en face surface aortic lesion area decreased by 20.45%, 13.64% and 24.5% in simvastatin, pinocembrin and combination therapy, respectively. There was a similar percentage of aortic area (lesion area compared to total aortic area) between the simvastatin and combination therapy.

Atherosclerotic lesion was also measured as the aortic root cross sectional lesion areas in mice (Figure 5C and E). Similarly, microscopic analysis of lesions stained with oil red O at the level of the aortic sinus showed that the lesions decreased by 25.85%, 18.37% and 34.78% in simvastatin, pinocembrin and the combination therapy groups, respectively, compared to vehicle-treated group. The cross-sectional lesion areas in the combination therapy were significantly smaller (by 45%) than those of the simvastatin single.

## Discussion

Hyperlipidemia is recognized as one of the most critical risk factors in the development of atherosclerosis. High fat diets have been used to induce hypercholesterolemia and atherosclerosis in ApoE^−/−^ mice. A major finding in this study is that the combination therapy with simvastatin and pinocembrin effectively reduces serum lipid, protects vascular endothelial cells from dietary cholesterol– induced dysfunction, and reduces atherosclerotic plaque progression in the ApoE^−/−^ mice. The benefits of simvastatin alone or in combination with pinocembrin on plaque size are associated with amelioration of vascular endothelial cells function. The potency and effectiveness of the combination were not less than that of simvastatin alone, although the dose of simvastatin was small in the combination group. This study demonstrated for the first time that pinocembrin was able to cooperate with simvastatin in inhibiting the progression of atherosclerosis in the ApoE^−/−^ mice and the combined effects are synergistic.

In recent years, propolis has been used extensively in food and beverages because it is thought to improve human health and to prevent diseases such as heart disease, diabetes and even cancer. Because of its broad spectrum of biological activities and uses in health food, there is a renewed interest in the active constituents and pharmacological mechanisms of propolis. Pinocembrin is one of the most abundant flavonoids in propolis and has been reported to have multiple actions such as anti-inflammatory, anti-oxidative and anti-apoptotic activities [18,19]. Zhu et al. [10] reported that pinocembrin induced relaxation of rat aortic rings through an endothelium-dependent pathway, while our results suggested that the combination of simvastatin and pinocembrin inhibited atherosclerotic lesion, and the effect may be partially dependent on the protecting vascular endothelium and anti-oxidative properties of pinocembrin.

Clinical trials have demonstrated that statins treatment inhibit the progression of atherosclerosis and reduce the frequency of acute coronary events and stroke, attributed to the lowering of circulating lipid concentrations. Interestingly, statins treatment can not affect lipid levels in rodents, even with a high dose statin [14,15]. The ApoE^−/−^ mouse is a well-established genetic mouse model of atherogenic hypercholesterolemia, even with normal diet which spontaneously develops atherosclerosis with similar features to those observed in human hyperlipoproteinemia, and high fat diet can accelerate atherosclerosis lesion formation [20]. Unlike other studies that employed a much higher dose of simvastatin (100 mg/kg/day, or 300 mg/kg/day) [14,15], the simvastatin dose (10 mg/kg/day) in the present study was substantially lower than many reported in the literature. Consistent with previous reports [13-15] that this dose did not significantly decrease lipid levels since the animals were fed a high fat diet, which greatly increased plasma cholesterol levels. However, the smaller dose of simvastatin (5 mg/kg/day) combinating with pinocembrin lowered blood lipid levels in apoE^−/−^ mice. There was no significant difference in lipid-lowering effect between combination therapy and the single pinocembrin, we thus speculated that this effect would be mainly mediated by pinocembrin in the combination therapy.

Dyslipidemia plays an important role in the vascular endothelial cells dysfunction. Endothelial function is impaired in experimental model of atherosclerosis, including apoE^−/−^ mice [15]. Impaired endothelium-dependent vasodilation is considered as the hallmark of endothelial dysfunction, which precedes the development of atherosclerosis. When the balance between vasoconstriction and vasodilation is upset, endothelial dysfunction occurs and subsequently initiates a number of processes that promote or exacerbate atherosclerosis. The maintenance of vascular homeostasis is accomplished by the release of numerous dilator and constrictor substances. NO mediates endothelium-dependent vasodilation by opposing the effects of endothelium-derived vasoconstrictors such as ET-1. A defect in NO production or activity has been proposed as a major mechanism of endothelial dysfunction and a contributor factor in the progression of atherosclerotic lesions. Previous studies [21] reported that simvastatin preserves endothelial function in experimental hypercholesterolemia in association with an increase in eNOS levels independent of cholesterol-lowering activity. Statins also prevent the enhanced vasoconstrictor response to ET-1 of aortas in ApoE^−/−^ mice [22]. Our results showed that the combination of simvastatin and pinocembrin increased the level of serum NO, reduced the levels of serum ET, and reversed unfavorable imbalance between NO and ET.

Hypercholesterolemia also results in oxidative stress and generates excessive reactive oxygen species (ROS) such as superoxide anions (·O_2_^-^). Under physiological conditions, ROS can modulate cell proliferation, apoptosis and gene expression through the activation of transcription factors, and excessive ROS can be rapidly scavenged by endogenous antioxidant defenses such as SOD [16]. But under pathological conditions, ·O_2_^-^ reacts with NO to form ·ONOO-, resulting in decreasing NO bioavailability [16,23]. In this study, we found that the combination of simvastatin and pinocembrin stimulated endothelial NO production compared to vehicle-treated group. We supposed that the restoring endothelium function mainly be dependent on antioxidant property of the combination, therefore MDA content and SOD activity in serum were determined. Notably, MDA content decreased and SOD activity increased in the combination therapy. Of course, the mechanisms by which the combination therapy exerts these effects on the vascular endothelial cells remain to be further evaluated.

Low NO bioavailability induces VEGF expression, which not only promotes normal and pathological angiogenesis but also plays an important role in atherosclerotic lesions. Increased VEGF levels have been reported in plasma from hypercholesterolemic individuals [24], in coronary arteries from hypercholesterolemic pigs [25] and in the vascular wall of ApoE^−/−^mice [17]. Compared to normal vascular endothelium, in the area of high VEGF expression, endothelial cell adhesion molecule expression is increased, vascular permeability is enhanced, and mononuclear cell and lipid infiltration to the subendothelial area is elevated [26]. In this study, we showed that increases in plaque area were accompanied by increased VEGF expression in ApoE^−/−^ mice. Simvastatin could reduce the expression of VEGF which was not dependent on the hypolipidemic effect [27]. Simvastatin combinating with pinocembrin not only reduced the plaque areas but also restrained the expression of VEGF in the ApoE^−/−^ mice. The striking benefit achieved with the combination treatment in the ApoE^−/−^ mice cannot be attributed to simvastatin effect alone, while the smaller dose of simvastatin was administered.

## Conclusion

In summary, the present study demonstrated that the combination treatment of simvastatin and pinocembrin for 14 weeks significantly decreased serum lipid levels, improved endothelial function and reduced atherosclerosis in ApoE^−/−^ mice, while the simvastatin had no prominent effect on serum cholesterol levels. Although the mechanism is not yet elucidated fully, further research may lead to new understanding of the actions of the combination and new therapeutic interventions for atherosclerosis.

## Abbreviations

apoE: Apolipoprotein E; ApoE^−/−^: Apolipoprotein E knockout; HDL-C: High density lipoprotein cholesterol; LDL-C: Low density lipoprotein cholesterol; TC: Total cholesterol; TG: Triglyceride; NO: Nitric oxide; ET: Endothelin; SOD: Superoxide dismutase; MDA: Malondialdehyde; ET: Endothelin; VEGF: Vascular endothelial growth factor; ROS: Reactive oxygen species; ·O_2_^-^: Superoxide anions.

## Competing interests

The authors declare that they have no competing interests.

## Authors' contributions

HS, NY, SY carried out the study, data collection and analysis and drafted the manuscript. FL was responsible for the preparation of pinocembrin. JW was responsible for the funding. YF carried out the biochemical analyses. SQ was responsible for the study design, the data analysis, and the drafting of the manuscript. All authors read and approved the final manuscript.
